# Fog collection improvement using harp structures formed from hairy yarns

**DOI:** 10.1038/s41598-025-28497-2

**Published:** 2025-12-29

**Authors:** Atieh Almasi, Hooshang Nosraty, Seyed Abolfazl Mirdehghan

**Affiliations:** Textile Engineering Department, Amirkabir University of Technology, Tehran, Iran

**Keywords:** Fog collector, Fog collection efficiency, Hairy yarn, Harp, Cotula fallax plant, Engineering, Materials science, Physics

## Abstract

With the increasing global problem of water scarcity, unconventional methods for water supply, such as fog collection, are emerging as viable solutions in certain regions. This study proposes a harp structure composed of hairy yarns, inspired by the Cotula fallax plant, to improve the fog collection efficiency. The presence of hair fibers can greatly enhance fog collection efficiency. In the harp structure, where yarns are widely spaced, thin hair fibers improve aerodynamic efficiency by increasing the shade coefficient without substantially affecting the pressure drop coefficient of the fog collector. Due to their extreme fineness, these fibers can increase droplet capture efficiency by approximately 2.5 times compare to the optimized Raschel mesh and reduce onset time. Harp structures formed by hairy yarns, owing to the entanglement of their hair fibers, retain more water on their surfaces than the optimized Raschel mesh, and the clogging of these structures is influenced by the arrangement of the hair fibers. The parallel arrangement of the hair fibers forms channels that transfer the water via capillary action, which reducing clogging. When the hair fibers are oriented downward, smaller droplets separate directly from fibers, further decreasing onset time. These findings offer a deeper understanding of the role of hair fibers in the fog collection process and propose an effective strategy for designing an optimal fog collector. Specifically, this study experimentally evaluates the impact of the fineness, arrangement, orientation, and density of hair fibers on fog collection efficiency.

## Introduction

The water crisis is one of the most important challenges facing humanity in the 21 st century. In addition to environmental issues, it has adversely impacted food security and global health^1^; Consequently, various methods for providing water to communities have been explored, including fog water harvesting^2^. This technique has been implemented in several regions around the world where dense fog is prevalent and access to water is scarce^3–6^. Fog harvesting technology can be regarded as a viable solution for providing water to these populations.

Currently, the most common method of fog collection involves installing fibrous meshes in the perpendicular path of fog flow. In this process, fog droplets pass through the mesh carried by the wind and are captured by the collector mesh fibers. The collected droplets grow by accumulating additional droplets and merging with one another. Once they reach a critical volume, the weight of the droplet overcomes the adhesion force between the droplet and the fiber, causing them to detach and fall into a water container for storage. Raschel meshes, made from polyethylene or polypropylene fibers with high UV resistance, are the most widely used fibrous structures in fog collection projects^6,7^.

Fibrous meshes have been extensively utilized in the fog collection process due to their simplicity, affordability, availability, and low costs associated with installation, repair, and maintenance, as well as their lack of energy requirements^3^. However, these meshes have certain limitations, including low efficiency^8^, a dependence of efficiency on wind speed^9^, and a reduction in collection efficiency due to clogging^10^. Researchers have sought to address these limitations and optimize fog collector meshes by investigating the effects of various parameters on fog collection efficiency. These parameters include fiber wettability, fiber cross-sectional shape, fiber fineness, fiber arrangement, fiber spacing within the mesh, and the geometry of the mesh unit cell^11–18^.

Inhabitants of arid regions often utilize specific structures to secure their water supply^19^. Various species of plants and cacti^20–22^, Namib Desert beetles^23,24^, and spider webs^25^ exemplify organisms with unique biological adaptations that enable them to harvest water and endure drought conditions. The remarkable capabilities of these natural structures have inspired researchers to develop new designs, including nature-inspired fibrous structures^26–30^, that address the limitations of conventional fibrous meshes used for fog collection^31–37^. While these innovative structures often demonstrate superior collection efficiency compared to traditional fog collectors, several challenges, such as high production costs, low durability, and insufficient strength under environmental conditions, as well as limitations in large-scale production, have hindered their widespread application in fog collection projects^34^.

In addition, to improve fog collection efficiency and reduce clogging in the collector mesh, researchers have proposed various methods, including replacing the fibrous mesh with a harp structure^38–41^ or hair brushes^42,43^, as well as applying electrostatic forces^44–46^.

In this article, inspired by the Cotula fallax plant^22^, we evaluate the impact of presence of hair fibers on fog collection efficiency. For this purpose, Cotula fallax is a native plant of desert regions that possesses unique characteristics for collecting fog droplets. The branches and leaves of this plant are covered with fine, long, flexible, and hydrophobic hairs that form a three-dimensional structure. The hair presented in the structure of Cotula fallax plant enhance droplet capture and can retain a large volume of fog droplets by wrapping around them. Given that harp structures are more suitable than fibrous meshes for the fog collection process^39,41,47–49^, this paper experimentally evaluates the performance of harp structures composed of hairy yarns by inspiring Cotula fallax plant. Unlike many other nature-inspired designs, these structures are affordable, can be produced on a large scale, and can be widely utilized in fog collection processes.

## Fog collection efficiency

The optimization of fibrous fog collector structures requires a comprehensive understanding of the fog collection process. The collection efficiency of a fog collector ($\:{\upeta\:}$) is directly proportional to its aerodynamic efficiency (η_ace_), droplet capture efficiency (η_cap_), and drainage efficiency (η_dra_)^50^:

1 $$\:{\upeta\:}={\eta\:}_{ace}{\eta\:}_{cap}{\eta\:}_{dra}$$

The aerodynamic efficiency is defined as the ratio of the flow entering the collector structure to the total flow passing through an equivalent surface area of the collector at a far field^50^:

2 $$\:{\eta\:}_{ace}=\frac{{v}_{1}}{{v}_{0}}S$$

where v_0_, v_1_ and S are fog flow velocity of close to the fog collector, unperturbed velocity of fog flow and shade coefficient, respectively. As aerodynamic efficiency increases, the ratio of the flow passing through the collector surface to the flow deviating from the collector structure also increases. The aerodynamic efficiency of a fog collector depends not only on the overall shape of the collector but also on the shade coefficient (cover factor) and the pressure drop resulting from the flow passing through the mesh. Therefore, the aerodynamic efficiency of a fog collector is^50^:

3 $$\:{\eta\:}_{ace}=\frac{S}{1+\sqrt{{C}_{0}/{C}_{d}}}$$

Where, S represents the shade coefficient, C_0_ denotes the pressure drop coefficient of the fog collector, and C_d_ signifies the drag coefficient of the collector, assuming it is impermeable. The shade coefficient (S) is a key parameter in fog collector design. In some cases, this parameter can be calculated using a mathematical model to facilitate the geometric design of the fog collector^51^.

The capture efficiency is defined as the ratio of the number of droplets collected by the fibers (N) to the total number of droplets passing through the fog collector (N_T_):

4 $$\:{\eta\:}_{cap}=\frac{N}{{N}_{T}}$$

The size of the fog droplets and the diameter of the fibers that constitute the fog collector determine that the most important mechanism for fog droplet capture is inertial impaction, while the effects of other capture mechanisms can be disregarded. In this mechanism, the efficiency of droplet capture is a function of the Stokes number, which is defined as^52^:

5 $$\:St=4\rho\:{r}^{2}V/\mu\:d$$

In this relation, ρ, r, V, µ, and d represent the density of water, radius of the fog droplets, velocity of the fog flow, viscosity of air, and diameter of the fibers, respectively. An increase in the Stokes number enhances the capture efficiency. Consequently, as stated in Eq. 5, increasing the velocity and diameter of the droplets, along with the fineness of the fibers, raises the probability of droplets collision to the fiber.

The drainage efficiency of a fog collector is defined as the ratio of the collected droplets in the water container to the total number of captured droplets. The fog collector must effectively transfer the captured droplets to the water container to prevent re-entrainment of droplets back into the fog flow and to minimize clogging phenomena. Retained droplets on the collector’s surface can not only alter the drainage efficiency but also impact the aerodynamic and capture efficiency by obstructing the flow of fog through the collector structure^38^. The drainage efficiency of a fog collector is influenced by the wetting properties and cross-sectional shape of the fibers, as well as the arrangement and spacing between the fibers.

## Materials and methods

In this study, the effects of the presence of hair fibers on the fog collection process have been investigated, inspired by Cotula fallax plant. For this purpose, four types of hairy yarns with distinct characteristics of hair fibers were utilized to prepare various harp structures, along with a Raschel mesh, which is a commonly used fog collector structure and was optimized in fiber fineness and spacing. Tables 1 and 2 provide the specifications of these hairy yarns and the optimized Raschel mesh.

**Table 1:** Hairy yarns specifications.

Final Sample Code	Hair specification		Harp specification			Yarn count	Code	Sample Type
	Code	Hair direction	S (%)	Y.S. (cm)	Code	(Nm)		
H1S1D1	D1	Upward	78.45	1	S1	85	H1	Yarn
H1S1D2	D2	Downward						
H1S3D1	D1	Upward	42.56	3	S3			
H1S3D2	D2	Downward						
H2S1D1	D1	Upward	91.83	1	S1	90	H2	
H2S1D2	D2	Downward						
H2S3D1	D1	Upward	49.79	3	S3			
H2S3D2	D2	Downward						
H3S1R	R	Randomly oriented	29.01	1	S1	125	H3	
H3S3R	R	Randomly oriented	14.66	3	S3			
H4S3D1	D1	Upward	23.71	3	S3	109	H4	

Y. S.: Yarn spacing

S: Shade coefficient

**Table 2:** Optimized Raschel mesh specifications.

Final Sample Code		Fabric direction	S (%)	W.P.C.	C.P.C.	Code	Sample Type
R1		Knitting direction	21.11	14	51	R	Raschel Mesh
R2		Perpendicular to the knitting direction					

C.P.C.: Course per Centimeter

W.P.C.: Wale per Centimeter

S: Shade coefficient

**Fig. 1:**
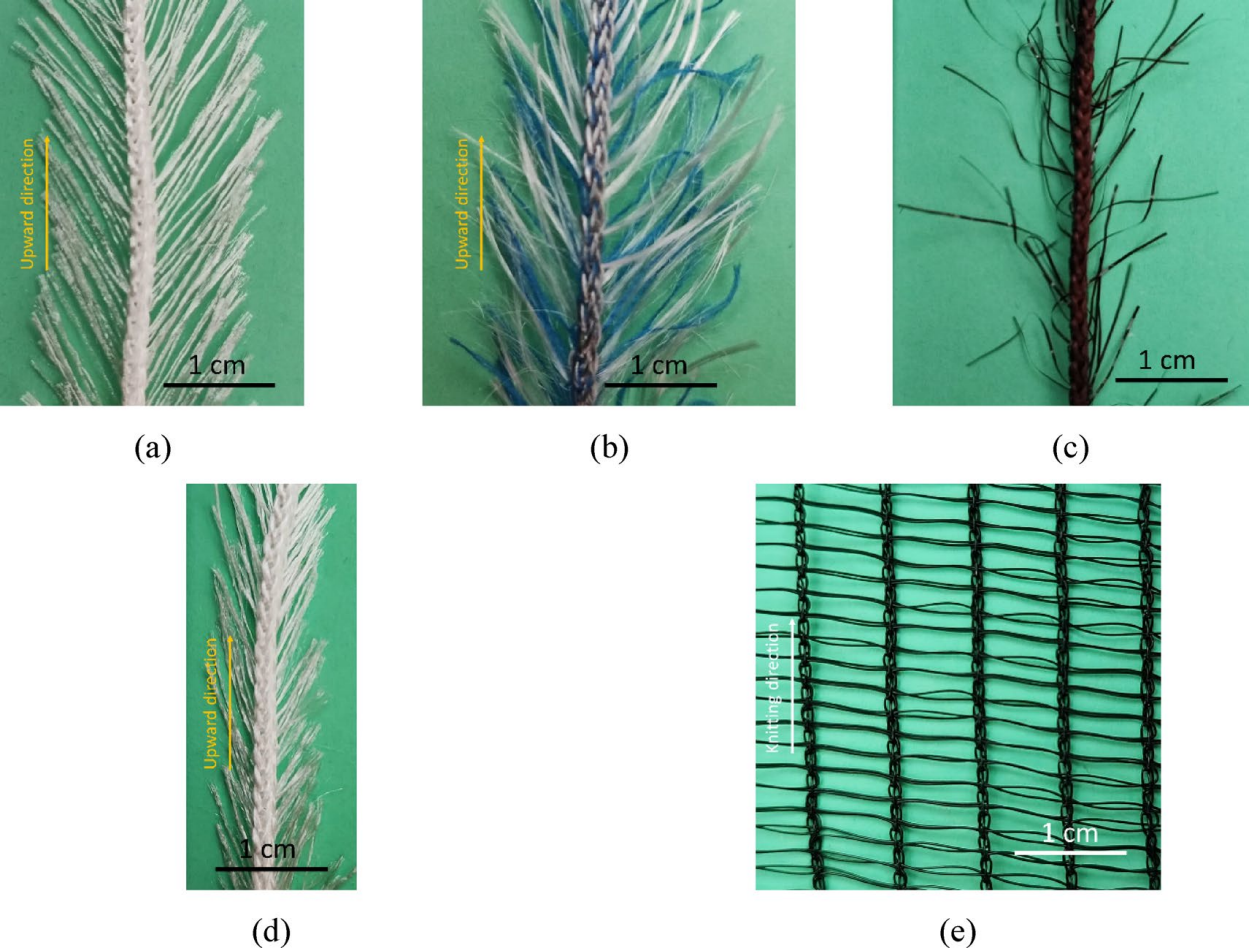
Examined samples: (a) H1, (b) H2, (c) H3, (d) H4, (e) R.

The images of all the samples are presented in Fig. 1 The hairy yarn samples vary in terms of their count and length, density, fineness, and arrangement of hair fibers. In the H1 samples (Fig. 1 (a)), the hair fibers are arranged into parallel groups at a specific angle to the core yarn. The H2 samples (Fig. 1 (b)) resemble the H1 samples; however, they exhibit a softer and denser appearance. This difference is attributed to the presence of fine individual hair fibers in the H2 samples, in addition to the parallel groups of hair fibers. These fibers, which are arranged at various angles and randomly, increase the density of hair fibers that led to the reduction in hair spacing. In the H3 samples (Fig. 1 (c)), all hair fibers are arranged individually at random angles to the core yarn. The density and average length of the hair fibers in this sample are lower than those in the H1 and H2 samples. The H4 sample (Fig. 1 (d)) is relatively similar to the H1 sample, except that the average length of the hair fibers is shorter. Figure 1(e) illustrates the R samples, which consist of a Raschel mesh optimized for fog collection.

**Fig. 2:**
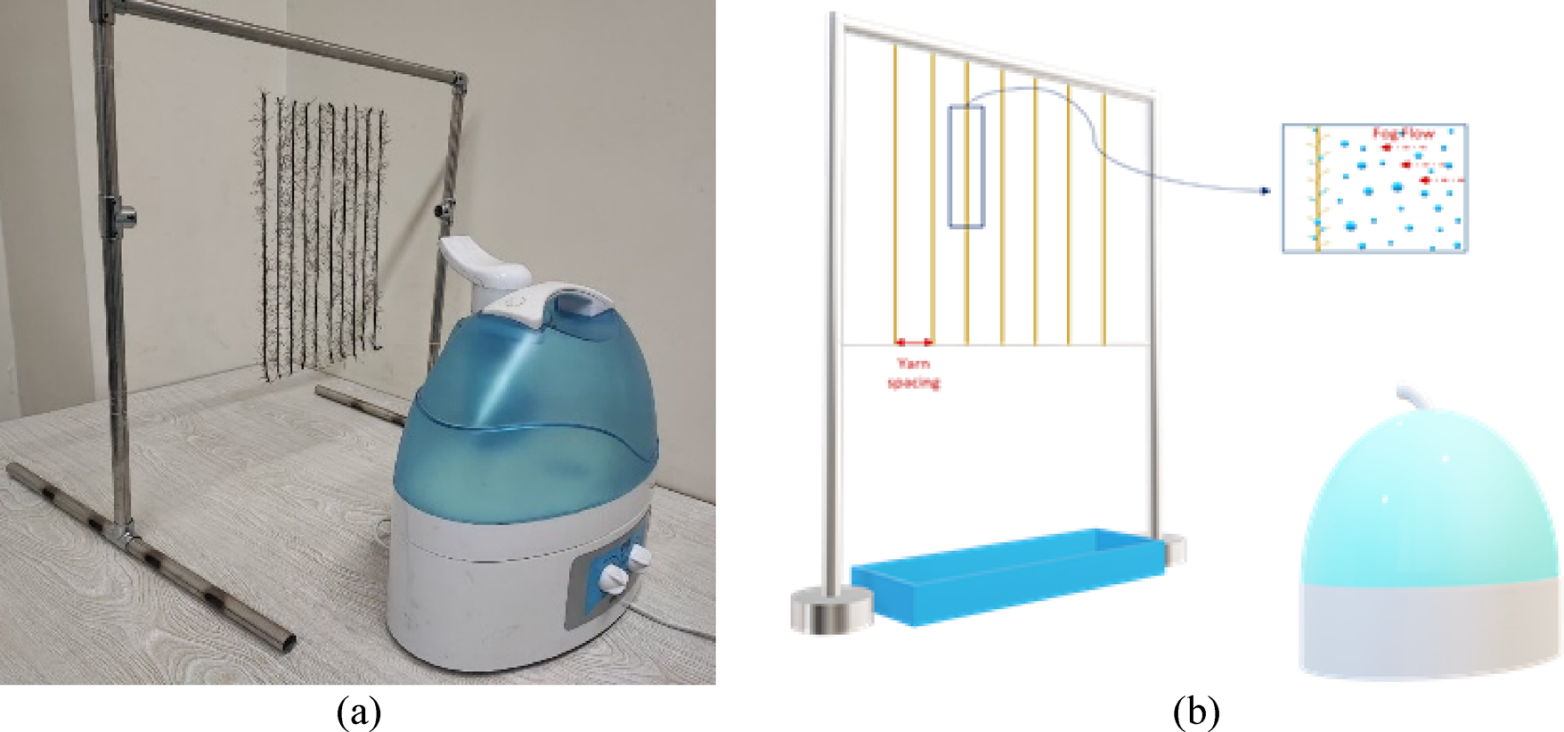
Experimental setup for fog collection (a) photograph and (b) schematic view.

Figure 2 presents a photograph and schematic representation of the setup utilized for the fog collection experiments. The hairy fog collectors were designed in the form of a harp structure composed of hairy yarns with varying yarn densities. Yarn density is inversely proportional to yarn spacing. The harp structures were constructed with two different yarn densities: at a higher density (S1), the hairy yarns were spaced 1 cm apart (samples H1S1, H2S1, and H3S1), while at a lower density (S3), the yarns were spaced 3 cm apart (samples H1S3, H2S3, and H3S3). Additionally, since the hair fibers in samples H1 and H2 have a specific orientation, both direction and density of the hairy yarns in the samples were altered. Samples were prepared with the hairy yarns oriented in both upward and downward directions (D1 and D2, respectively). The hair fibers in samples H1S1D1, H1S3D1, H2S1D1, and H2S3D1 are arranged upward, while in samples H1S1D2, H1S3D2, H2S1D2, and H2S3D2, the hair fibers are oriented downward.

Fog collection experiments were conducted under fog conditions in a chamber maintained at 25 °C and 75% relative humidity (RH). Fog droplets were generated using an ultrasonic humidifier, the Konfor model SPS-828 A, which has a droplet production capacity of 300 ml/h. All samples, measuring 30 cm × 30 cm, were mounted on an experimental base and positioned 15 cm away from the humidifier to allow exposure to the fog flow for 60 min. For each sample, the time required for the first droplet to fall and initiate the fog collection process, referred to as the onset time, was recorded, along with the weight of the collected water in the gutter every 5 min and the weight of the retained water on the samples.

All experiments were repeated three times on the samples to ensure the repeatability and validity of the results. Statistical analysis was conducted at a 0.05 confidence level to compare the means between groups.

## Results and discussion

In fibrous fog collector structures, enhancing collection efficiency is of paramount importance. This research investigates, for the first time, the use of Cotula fallax-inspired harp structures composed of hairy yarns to improve collection efficiency. The study examines the effects of the arrangement and density of hair fibers on the fog collection process. The results are compared with those obtained from the optimized Raschel mesh, which is a commonly used structure for fog collection.

### Effects of hair fibers on aerodynamic characteristics of fog collectors

The aerodynamic characteristics of a fog collector significantly influence the volume of fog flow that passes through its structure. Therefore, enhancing aerodynamic efficiency greatly impacts the amount of water collected. The presence of hair fibers in a fog collector affects aerodynamic efficiency in various ways, depending on the density, arrangement, and physical properties of the hair fibers. When the spacing between the hairy yarns in the harp structure is large or the hair fibers are sparse, the fog flow passing through the collector causes these fibers to deform. As a result, the presence of hair fibers has a negligible effect on the pressure drop coefficient of the fog collector^53^. The flexibility and fineness of the hair fibers mean that their presence on the collector structure does not significantly affect aerodynamic efficiency and shade coefficient is small enough that can be assumed η_ace_ ≈ 1.

As the density of hairy yarns in the harp structures increases, the entanglement of hair fibers in adjacent yarns due to the motion of fog flow also increases, resulting in an increase in shade coefficient and a decrease in the permeability of the collector structure. In this scenario, the presence of hair fibers elevates the pressure drop coefficient^54^ and diminishes aerodynamic efficiency.

Previous research has studied aerodynamic efficiency using analytical and numerical methods^50,55,56^, while experimental methods have been less commonly employed^57^. In this paper, the impact of hair fibers on aerodynamic efficiency has not been studied quantitatively and a theoretical analysis based on Eqs. 1 and 3, combined with experimental results, is applied to evaluate the effects of hair fibers on aerodynamic efficiency. Each harp sample was prepared using similar hairy yarns at two spacing distances: 1 cm and 3 cm. The shade coefficients of these samples, along with an optimized Raschel mesh in the absence of fog flow, were calculated using image processing techniques with the Fiji application. The results are presented in Tables 1 and 2.

**Fig. 3:**
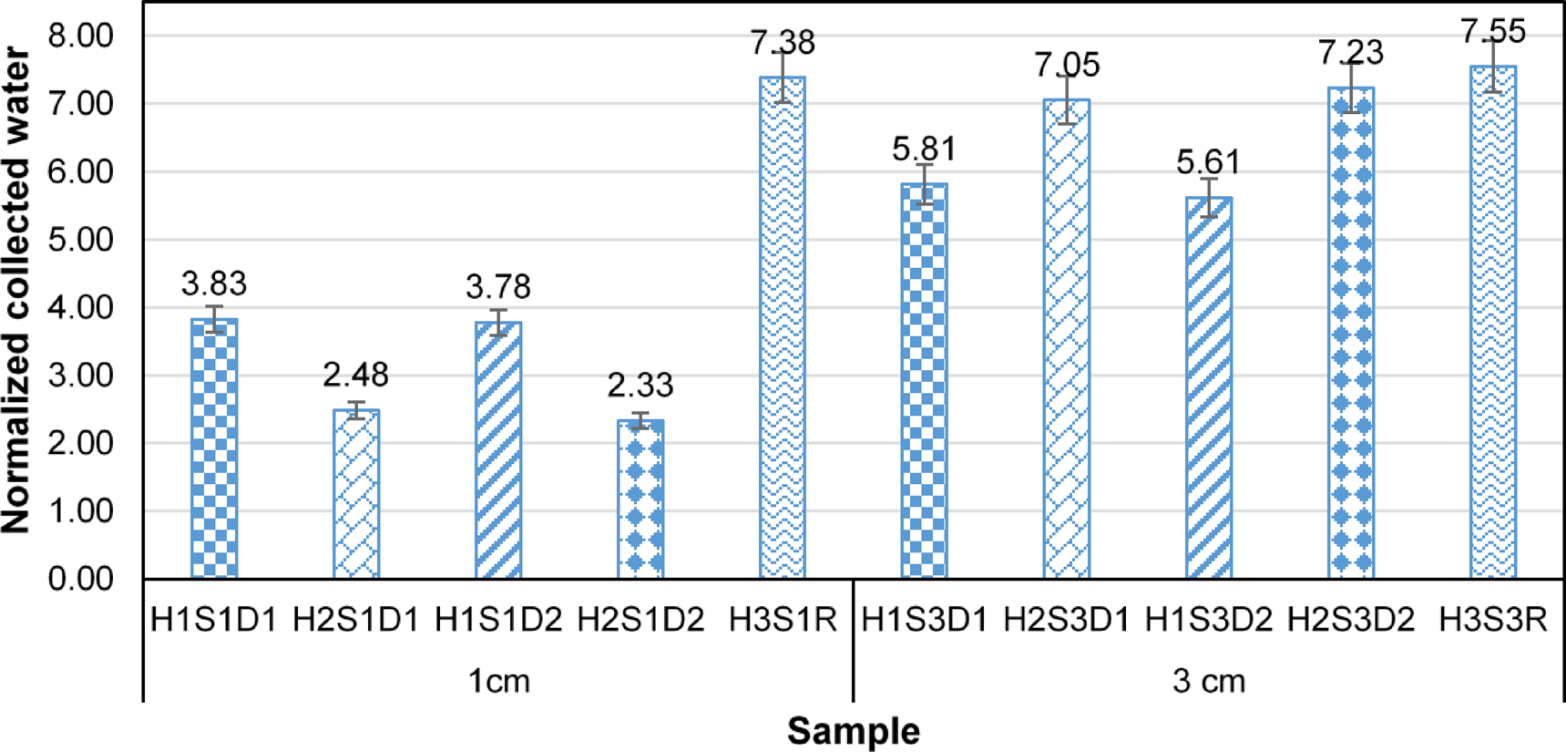
Effect of yarn spacing on total collected water.

Figure 3 shows the amount of water collected from these samples. The experimental data indicate that, with the exception of the H3S1R and H3S3R samples, a greater volume of water is collected at lower yarn densities. This phenomenon occurs because, at low yarn and hair densities, the shed coefficient of the collector structure is small, resulting in η_ace_ ≈ 1, and Eq. (1) changes to:

6 $$\:{\upeta\:}={\eta\:}_{cap}{\eta\:}_{dra}$$

According to Eq. (2), as yarn density increases and the shade coefficient exceeds its optimal range (between 0.5 and 0.6), the volume of fog flow passing through the collector decreases. Consequently, a larger portion of the flow is diverted away from the collector, following a peripheral path.

Statistical analyses were conducted to examine the effects of the yarns distance on the ration of collected water to each sample weight (normalize collected water) in H1, H2, H3 samples. The effect of the yarn spacing was significant for H1 and H2 (*p* < 0.05). In contrast, the effect of the yarn spacing was not statistically significant for H3 (*p* = 0.12). The average of normalized collected water for H1S3 (5.72 ± 0.14) to H1S1 (3.80 ± 0.06) and H2S3 (7.14 ± 0.15) to H2S1 (2.40 ± 0.09) are 1.5 and 2.98, respectively. In sample H2, the hair fibers exhibit a higher density compared to those in samples H1. Consequently, reducing the yarn spacing from 3 cm to 1 cm causes the shade coefficient of H1 and H2 to exceed the optimal range and increases the pressure drop coefficient. This results in a decrease in η_ace_ from its maximum value and reduces the total amount of collected water. In contrast, in sample H3, where the hair fibers are sparser and shorter, decreasing the yarn spacing has no significant effect on the aerodynamic efficiency, and the normalized collected water remains the same in both samples.

The aerodynamic properties of fog collectors significantly influence the overall fog collection process and are considered a limiting factor in enhancing collection efficiency. Therefore, understanding the impact of hair fibers within the fog collector structure on aerodynamic efficiency is crucial. Future studies should investigate in greater detail the effects of hair fiber characteristics—such as length, fineness, cross-sectional shape, flexibility, and orientation—on aerodynamic efficiency.

### Effects of hair fibers on capture efficiency

The presence of hair fibers in the fog collector structure, due to their high fineness, increases the Stokes number (Eq. 4) and the droplet capture efficiency. Additionally, it enhances the effective collection surface, thereby increasing the probability of droplet collisions with the fibers and improving capture efficiency.

**Fig. 4:**
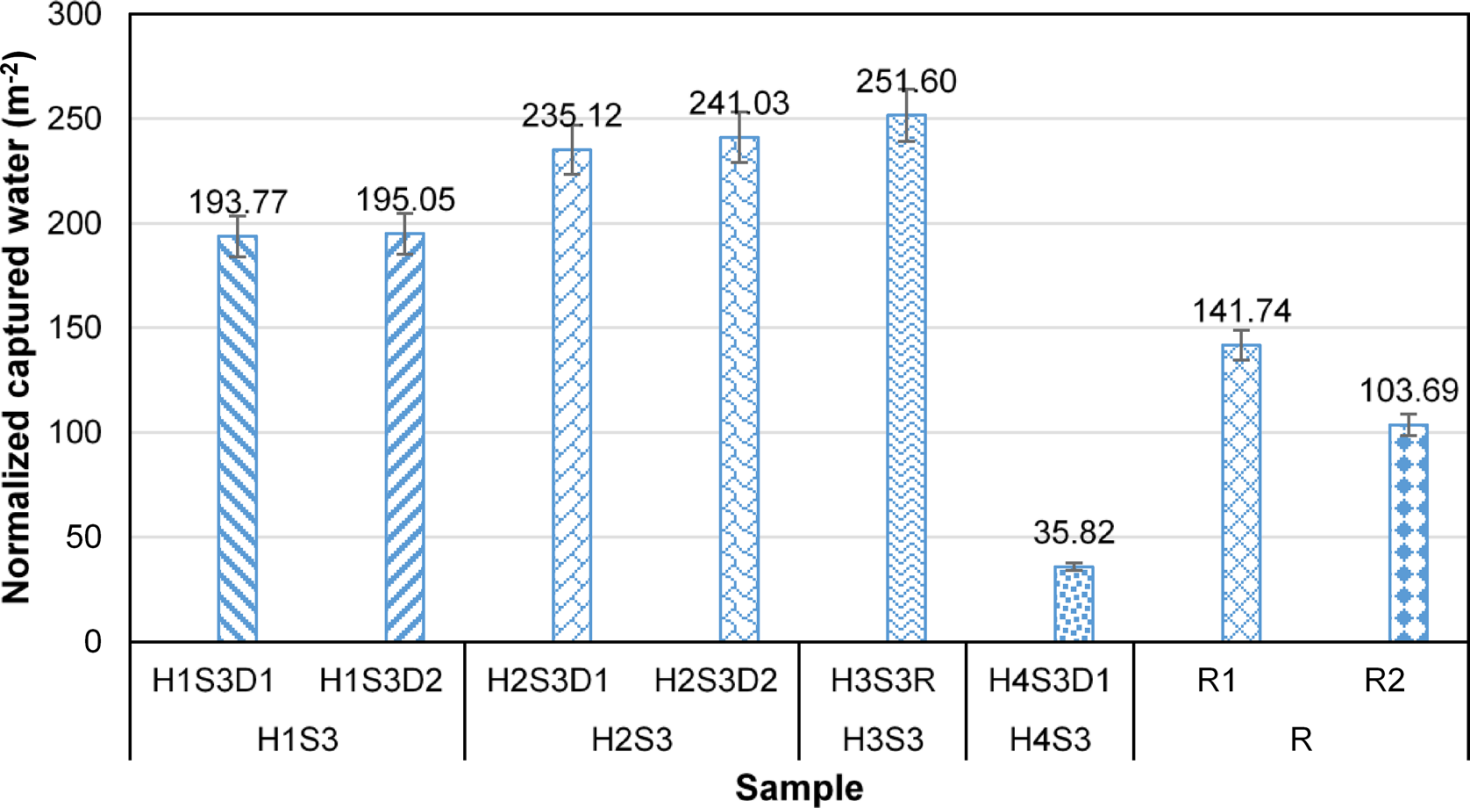
Normalized captured water per unit area of sample.

When the entire fog flow passes through the fog collector structure without any deviation, i.e., η_ace_ ≈ 100%, the amount of water collected by a fog collector can be considered a measure of its fog capture efficiency. Figure 4 illustrates the weight of captured droplets to the weight (normalized captured water) of each sample per unit area, in a scenario where the yarn spacing is sufficiently large to achieve η_ace_ ≈ 100%. According to this figure, all harp samples composed of hairy yarns exhibited higher capture efficiency than the optimized Rachel meshes, due to the presence of fine hair fibers. Among these, the H3S3R sample demonstrated the highest droplet capture, with 83.87 m^−^², which is approximately 1.77 times greater than the droplets captured by R1 and 2.43 times greater than those captured by R2.

To determine the effect of direction of H1S3, H2S3 and R on normalized captured water, statistical analyses were performed. The results indicate that sample direction has no significant influence on normalized captured water of H1S3 and H2S3. However, the difference between R1 and R2 was significant (*p* < 0.05) and can be attributed to the clogging phenomenon.

The normalized captured water of the H1S3 samples is, on average, 22.44% and 29.42% lower than that of the H2S3 and H3S3R samples, respectively. In the H1S3 samples, droplet capture results in the formation of bundles of hair fibers with a larger diameter than that of a single hair fiber, which reduces the droplet capture efficiency.

In the H4S3D1 sample, the formation of hair fiber bundles, combined with the shorter length of its hair fibers and a lower shade coefficient compared to the H1S3 samples, results in the lowest capture efficiency observed among the samples.

In the H2S3 samples, a similar reduction in capture efficiency occurs due to the formation of fiber bundles; however, the presence of individual hair fibers alongside these bundles increases the average droplet capture efficiency by approximately 25% compared to the H1S3 samples.

This trend in capture efficiency is also observed at shorter distances between hair yarns in harp structures, where the aerodynamic efficiency is less than 100%. However, because the density and arrangement of the hair fibers in various samples differ from one another, the ratio of fog flow through the collector structure and the variations in their aerodynamic efficiency also differ. Consequently, the weight of the collected water will not correspond to the fog capture efficiency.

### Effects of hair fibers on drainage efficiency

The droplet transport phenomenon in fog collector structures is a critical issue. These structures must efficiently transport droplets to prevent clogging and to detach captured droplets from the collector in the shortest possible time, thereby avoiding re-entrainment. Consequently, when evaluating drainage efficiency, three factors should be considered: the volume of retained water on the collector surface, the onset time and collection rate.

#### Retained water

**Fig. 5:**
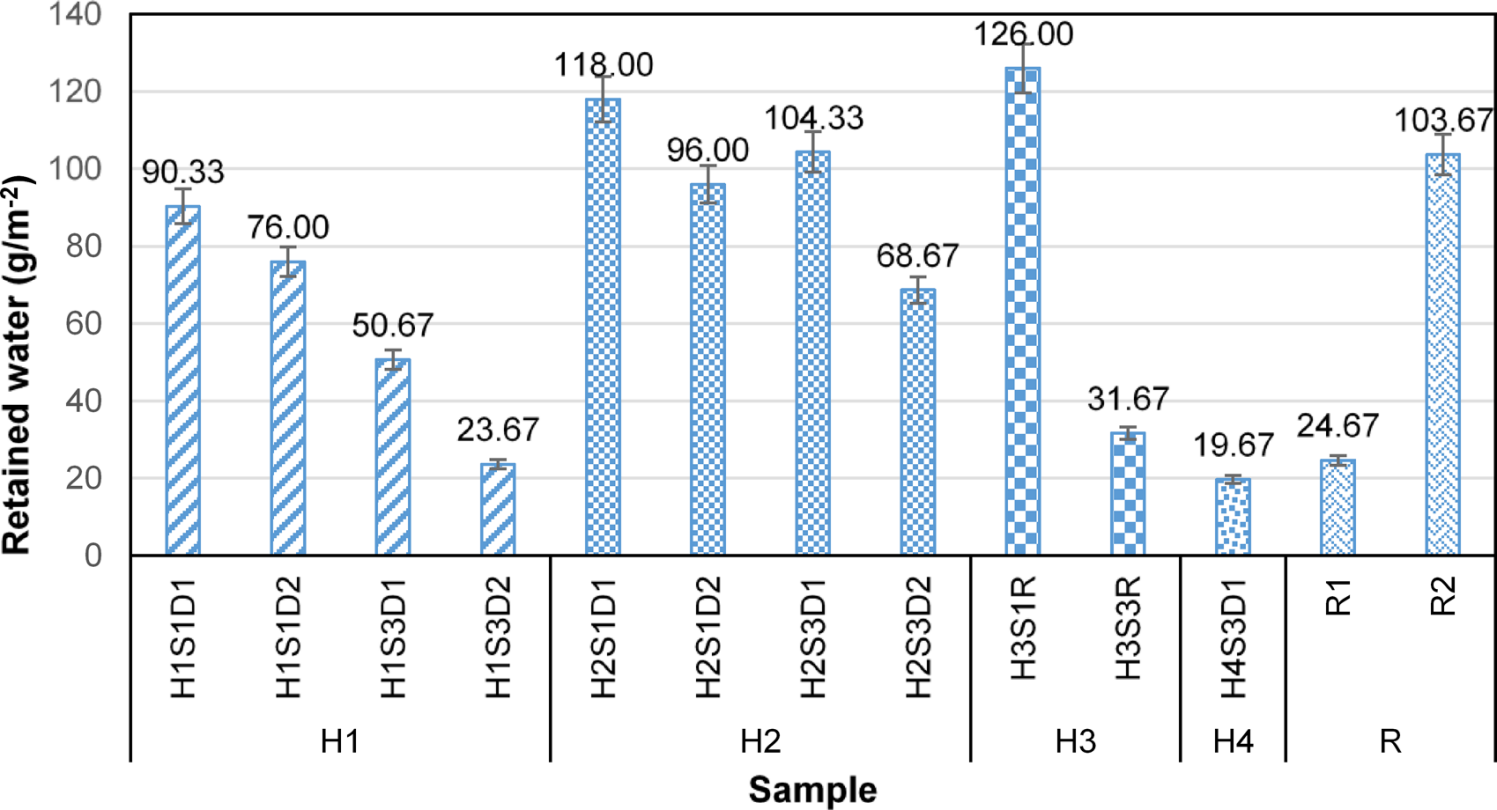
Retained water on a unit area of the sample.

The volume of trapped water on the fog collector can be considered an indicator of its clogging issues. Figure 5 illustrates the amount of retained water in each sample per square meter of mesh surface area. According to this figure, the harp structures, composed of hairy yarns, despite their favorable aerodynamic and capture efficiency, retain a larger volume of water on their surface compared to the Rachel mesh in the knitting direction (R1), generally. Specifically, the amount of retained water on the harp samples varied between 19.67 g/m² and 126.00 g/m², while the Rachel meshes retained between 24.67 g/m² and 103.67 g/m². In other words, R1 demonstrated superior performance in droplet transfer compared to the harp structures made of hairy yarns.

A statistical analysis of retained water in different samples indicates that both the direction and yarn distance have significant effects on the amount of retained water (*p* < 0.05). A droplet transport examination of various samples shows that the length, density, arrangement, and orientation of hair fibers are the most significant factors influencing the drainage behavior. Generally, in all harp samples, as the yarn spacing decreases, the amount of retained water in the samples increases. This is attributed to the fact that an increase in the density of hairy yarns enhances the entanglement of pile fibers and the intersection points of hair fibers, which can hold a greater volume of droplets.; in H2S1D2, it is about 1.40 times that of H2S3D2; and in H3S1R, it is roughly 4.00 times that of H3S3R. Furthermore, altering the orientation of samples H1 and H2, affected the amount of retained water.

In H1 samples, parallel groups of hair fibers upon contact with fog droplets, connect to one another and form distinct bundles of fibers. Within the inter-fiber spaces of these bundles, small channels are created that facilitate the transport of droplets. The captured droplets penetrate these channels through capillary action. As droplets penetrate the fiber bundles, they become less visible on the surface of the fibers, and the change in shade coefficient and permeability of the H1 samples resulting from droplet capture is expected to be negligible.

**Fig. 6:**
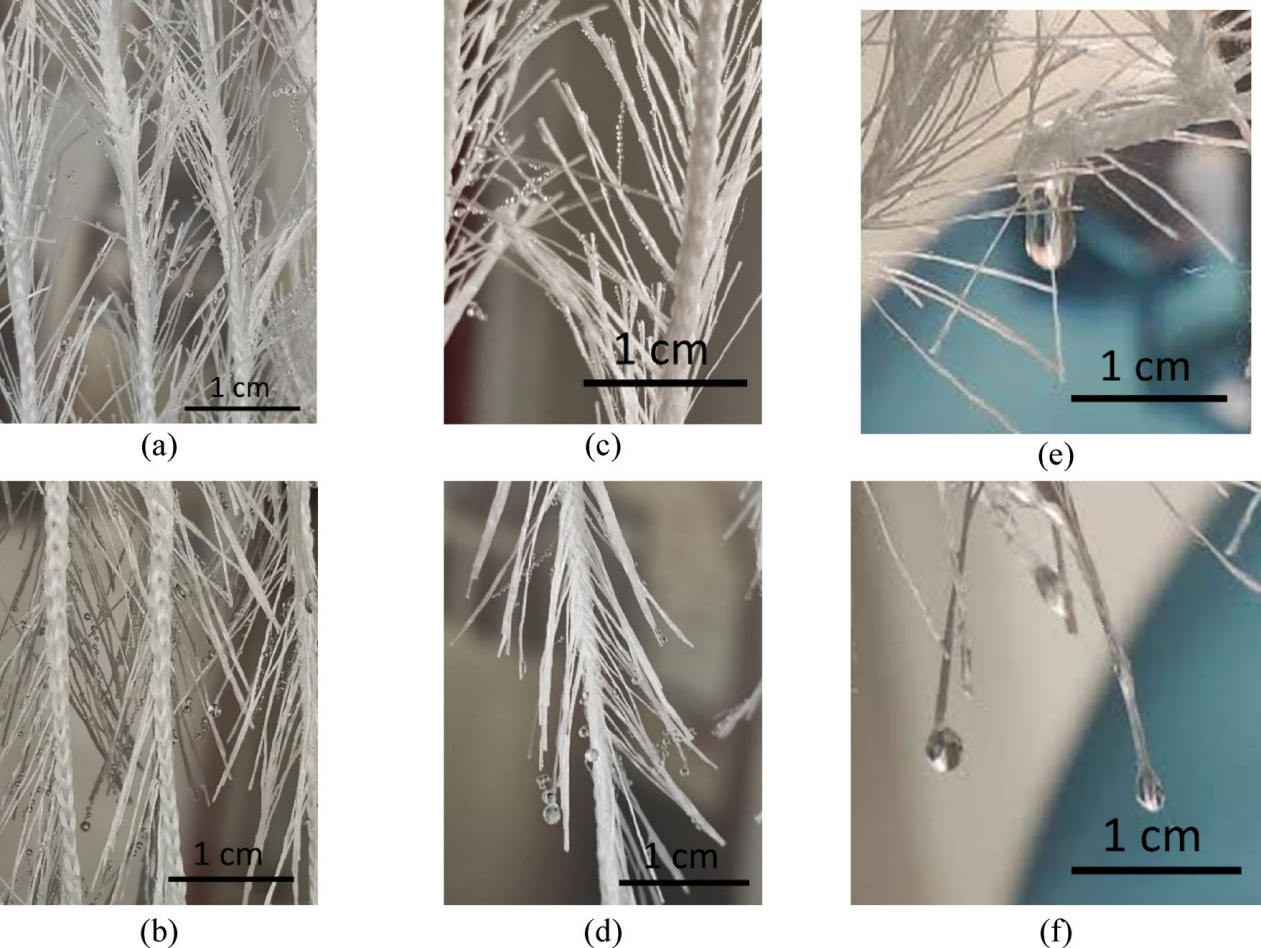
Fog collection process in H1 samples (a) H1S1D1 (b) H1S1D2 (c) H1S3D1 (d) H1S3D2 (e) detached droplets from core yarn (f) detached droplets from hair fibers.

Figure 6 shows the H1 samples during the fog collection process. In these samples, the orientation of the hair fibers, in addition to the inter-fiber water transfer channels, significantly influences droplet transfer. In H1S1D1 and H1S3D1, where the hair fibers are oriented upward, most of the captured droplets are directed toward the core yarn via the inter-fiber channels (Fig. 6 (a) and (c)). Conversely, in H1S1D2 and H1S3D2, where the fibers are oriented downward, the droplets are transferred to the ends of the fiber bundles without reaching the core yarn, resulting in their separation from the hair fiber bundles (Fig. 6 (b) and (d)). The finer and fewer fibers in the fiber bundles of the H1 samples, compared to the core yarn, lead to the detached droplets from H1S1D2 and H1S3D2 being smaller in size than those from H1S1D1 and H1S3D1, as depicted in Fig. 6 (e) and (f). Furthermore, the average amount of retained water in the H1 sample in the upward direction of the hair fibers (H1S1D1 (90.33 ± 1.53) and H1S3D1 (50.67 ± 0.66)) is approximately 1.41 times greater than that in the downward direction (H1S1D2 (76.00 ± 1.20) and H1S3D2 (23.67 ± 0.34)). The effect of yarn distance on retained water is statistically significant (*p* < 0.05), with the average retained water in H1S1 being approximately 2.24 times that of H1S3.

H4 sample exhibited water transfer behavior similar to that of the H1 samples; however, its shorter fiber length resulted in amount of retained water retention to be minimum.

**Fig. 7:**
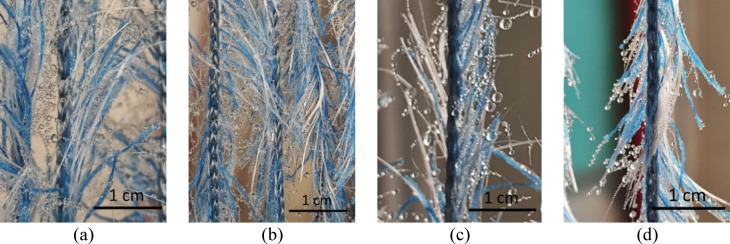
Fog collection process in H2 samples (a) H2S1D1 (b) H2S1D2 (c) H2S3D1 (d) H2S3D2.

In the H2 samples, the droplet transport behavior through the fiber bundles resembles that of the H1 samples; however, the individual hair fibers create a network on the yarn surface that can retain a significant volume of droplets. As shown in Fig. 7, the formation of this fibrous network in the H2 samples results in a greater volume of retained water and an increased clogging phenomenon compared to the H1 samples.

Statistical analysis shows that changes in the direction of the H2 samples and yarn spacing have a significant effect on the amount of retained water (*p* < 0.05). When the sample direction changes from downward (H2S1D2 (96.00 ± 1.76) and H2S3D2 (68.67 ± 1.20)) to upward (H2S1D1 (118.00 ± 1.46) and H2S3D1 (104.33 ± 1.60)), the average amount of retained water increases by 1.35 times. Additionally, decreasing the yarn spacing from 3 cm to 1 cm increases the average amount of retained water by 1.24 times.

**Fig. 8:**
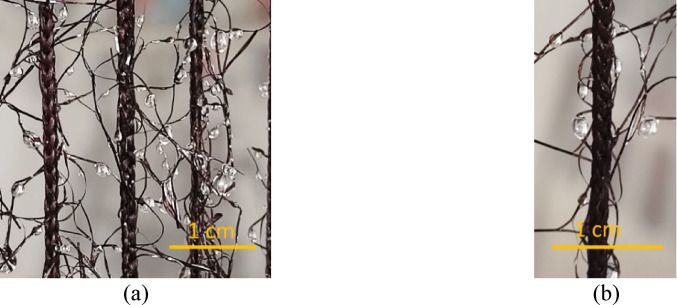
Fog collection process in H3 samples (a) H3S1R (b) H3S3R.

Figure 8 illustrates the H3 samples during the fog collection process. The droplet transport in the H3 samples, because of the single arrangement and random orientation of the hair fibers, differs from that in the other samples. In the H3 samples, the growth of captured droplets due to merging with other droplets occurs in areas of the yarn where hair fibers are closely spaced or where the droplet size is large. In these regions, droplets are held by two or more hair fibers until they reach their critical volume. Conversely, in areas with low hair fiber density or smaller droplets, individual droplets adhere to a single fiber. As the droplets enlarge, their weight displaces the hair fibers, bringing the droplets into contact with additional fibers. This process continues until the droplets reach their critical volume, at which point they detach from the hair fibers and are transferred to the water container. This droplet transfer behavior in H3 samples results in the amount of retained water in H3S1R (126.00 ± 1.30) being 3.98 times greater than that in H3S3R (31.67 ± 0.35).

Furthermore, the angle of each individual hair fiber relative to the core yarn determines the droplet transfer path. In hair fibers oriented nearly vertically, the enlargement of droplets causes them to flow over the hair fibers toward the core yarn, from which they are subsequently transferred to the water container. In other hair fibers, the growth of the droplets moves them toward their tips, and droplets are directly separated from the hair fibers.

The analysis of samples H1, H2, and H3 reveals that the harp structures composed of hairy yarns exhibit distinct characteristics regarding droplet transport and clogging phenomena. These variations depend on the density, arrangement, and orientation of the hair fibers. In Raschel meshes knitted from monofilament fibers, these characteristics are influenced by the density, angles, and orientation of the monofilaments^10,11^.

**Fig. 9:**
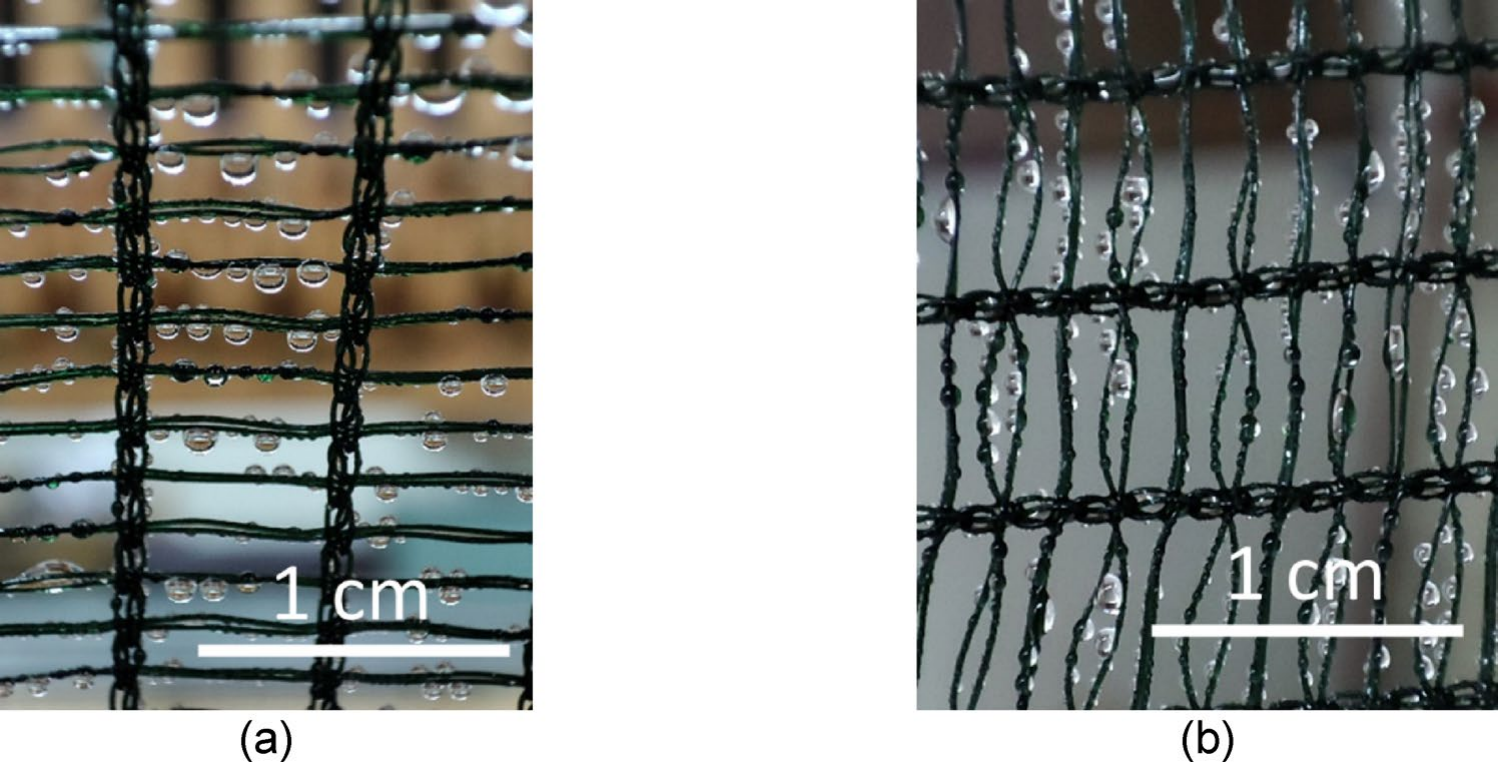
Fog collection process in Raschel meshes (a) R1 (b) R2.

Figure 9 shows the Raschel meshes during the water collection process. The orientation of the fog collector significantly impacts droplet transport and clogging within the Raschel meshes; for instance, the volume of retained water on the Raschel mesh aligned with the knitting direction (R1) is approximately 76% less than that on the Raschel mesh, oriented perpendicular to the knitting direction (R2). Consequently, the geometric arrangement of the fibers in Raschel meshes plays a crucial role in their interactions with water droplets, specifically in terms of droplet transfer and the retention of water on the fibers. This factor should be taken into account when studying drainage efficiency of the collector meshes.

#### Onset time and fog collection rate

One of the most important features of fog collector structures is their ability to efficiently and rapidly transport collected droplets. This capability not only enhances droplet transfer efficiency by preventing droplets from re-entering the fog flow but also facilitates fog extraction in situations where fog duration is short. Additionally, prolonged droplet retention on the surface of the collector can lead to clogging, which adversely affects both aerodynamic and capture efficiency^58^.

**Fig. 10:**
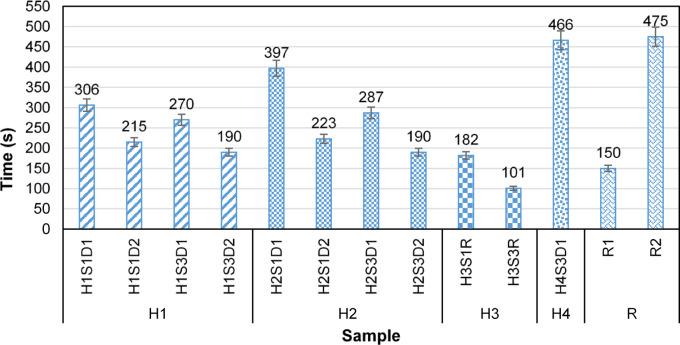
Onset time.

Figure 10 illustrates the time required for the first drop to fall from the fog collector structure across the various samples. This duration is regarded as the onset time for the fog collection process. The results indicate that the average time for the first drop to fall is 257 s for the harp composed of hairy yarns and 212 s for the Rachel meshes. The maximum onset time recorded is 475 s, associated with sample R2 (Raschel meshes oriented perpendicular to the knitting direction). This suggests that harp structures with hairy yarns are more effective than Raschel meshes regarding the onset time of the fog collection process.

According to Fig. 10, the density and arrangement of hair fibers, along with the orientation of the samples, are the key factors influencing the onset time in all harp structures. Statistical analyses reveal that the effect of hair density on onset time is significant (*p* < 0.05). An increase in hair density results in a longer onset time; as hair density increases, the entanglement of fibers and the number of intersecting points where fog droplets accumulate also rise.

Furthermore, the results of the statistical analysis indicate that changes in the direction of the samples have a significant effect on the onset time (*p* < 0.05). The changes in the direction of hair fibers in the H1 and H2 samples result in variations in the forces acting on the droplets and the size of the detached droplets, which affect the onset time.

According to the graph, among the harp samples composed of hairy yarns, H3S3R exhibited the shortest onset time at 101 s, followed by H3S1R at 182 s. Both H2S3D2 and H1S3D1 shared the third position with an onset time of 190 s. The average onset time increased in the H3, H1, and H2 samples, respectively. In the H3 samples, the presence of individual hair fibers within the yarn contributed to the shortest onset time. This is attributed to the fineness of these fibers, which enhances collection efficiency and droplet growth rate while reducing the critical volume of droplets detached from the fibers. The synergistic effect of increased capture efficiency and decreased critical volume of droplet ultimately leads to a reduction in onset time.

In H1 samples, the formation of fiber bundles creates water transfer channels, which reduces capture efficiency. Additionally, the captured droplets can detach from the fog collector after filling the empty spaces between the fiber channels. These parameters result in a longer separation time for the first droplet in H1 samples compared to H3 samples. These factors maximized the onset time of H4S3D1.

In the H2 samples, the increased density of hair fibers, resulting from the presence of individual fibers adjacent to parallel groups, leads to the higher number of crossing points and a longer onset time compared to the H1 samples.

In R samples (Raschel meshes), the onset time is highly dependent on the direction of mesh installation. Consequently, sample R1 exhibits the best performance in terms of droplet fall speed, following sample H3S3R, while sample R2 has the longest onset time among all samples.

In addition to onset time, the fog collection rate is another important factor affecting the performance of the fog collection process. The fog collection rate represents the discharge rate of the droplets captured by the collector structure and corresponds to the slope of the volume of collected water over time after reaching steady-state conditions. From the start of the collection process until steady state is achieved, the fog collection rate is not constant and varies over time. Unlike onset time, which is significantly influenced by both yarn density and sample orientation, yarn spacing is the sole factor affecting the fog collection rate under steady-state conditions.

**Fig. 11:**
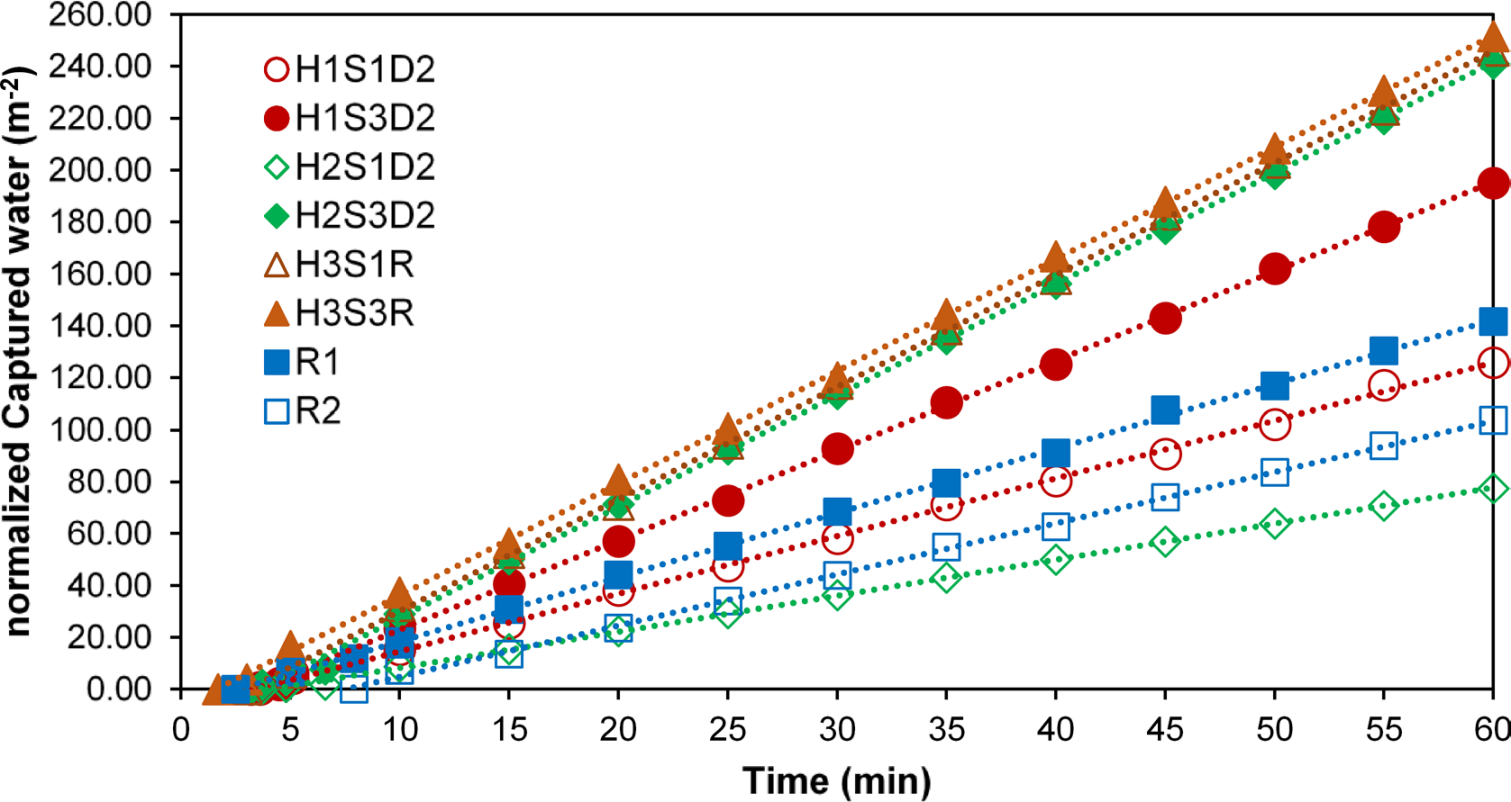
Fog collection rate.

Figure 11 shows the fog collection rate of different examined samples and indicates that samples with lower hair fiber densities have higher collection rates compared to those with higher densities. The H3 sample exhibits the highest fog collection rate, while the H2S1D2 sample has the lowest rate among all samples. This variation in collection rate can be attributed to the density and arrangement of the hair fibers.

## Conclusion

In this paper, the effects of hair fibers on fog collection efficiency, inspired by Cotula fallax plant, were investigated. Generally, the presence of hair fibers in fog collector structures can significantly enhance fog collection efficiency, depending on the density and arrangement of the hair fibers, compared to optimized Raschel mesh. Research indicates that when the yarn density in the harp structure is low, the presence of hair fibers improves aerodynamic collection efficiency by increasing the cover factor. Furthermore, due to their high fineness, presence of hair fibers in the harp structure can boost capture efficiency by approximately 2.5 times that of optimized Raschel mesh. In harp structures, the critical volume of droplets that detach directly from the hair fibers is smaller than that of other droplets, and these droplets remain on the hair fibers for a shorter period. However, as the density of hair fibers increase or they become closer, droplets are held by two or more fibers, resulting in an increased volume of retained droplets. This phenomenon causes optimized Raschel mesh to exhibit better drainage characteristics compared to harp structures composed of dense hairy yarns. Additionally, hair fibers arranged in parallel bundles can hold a smaller volume of droplets and experience less clogging due to the transfer of collected water through liquid channels formed between the fibers. In contrast, a random arrangement of hair fibers and a reduction in their spacing can decrease drainage efficiency and exacerbate clogging. Moreover, the angle of the hair fibers relative to the yarn axis affects droplet transfer and onset time. When the hair fibers are oriented downwards, the critical volume of droplets is reduced, allowing droplets to be transferred to the container faster without passing through the central yarn. A change in the direction of the hair fibers from downward to upward can increase the amount of retained water by more than twofold and decrease the onset time by at least 80 s.

## Data Availability

The research data and material are available from the corresponding author on reasonable request.
